# ^1^H Magnetic Resonance Spectroscopy to Understand the Biological Basis of ALS, Diagnose Patients Earlier, and Monitor Disease Progression

**DOI:** 10.3389/fneur.2021.701170

**Published:** 2021-08-27

**Authors:** Sarah Caldwell, Douglas L. Rothman

**Affiliations:** Departments of Radiology and Biomedical Engineering, Magnetic Resonance Research Center, Yale University School of Medicine, New Haven, CT, United States

**Keywords:** amyotrophic lateral sclerosis, magnetic resonance spectroscopy, glutamate, GABA, biomarker

## Abstract

At present, limited biomarkers exist to reliably understand, diagnose, and monitor the progression of amyotrophic lateral sclerosis (ALS), a fatal neurological disease characterized by motor neuron death. Standard MRI technology can only be used to exclude a diagnosis of ALS, but ^1^H-MRS technology, which measures neurochemical composition, may provide the unique ability to reveal biomarkers that are specific to ALS and sensitive enough to diagnose patients at early stages in disease progression. In this review, we present a summary of current theories of how mitochondrial energetics and an altered glutamate/GABA neurotransmitter flux balance play a role in the pathogenesis of ALS. The theories are synthesized into a model that predicts how pathogenesis impacts glutamate and GABA concentrations. When compared with the results of all MRS studies published to date that measure the absolute concentrations of these neurochemicals in ALS patients, results were variable. However, when normalized for neuronal volume using the MRS biomarker N-acetyl aspartate (NAA), there is clear evidence for an elevation of neuronal glutamate in nine out of thirteen studies reviewed, an observation consistent with the predictions of the model of increased activity of glutamatergic neurons and excitotoxicity. We propose that this increase in neuronal glutamate concentration, in combination with decreased neuronal volume, is specific to the pathology of ALS. In addition, when normalized to glutamate levels, there is clear evidence for a decrease in neuronal GABA in three out of four possible studies reviewed, a finding consistent with a loss of inhibitory regulation contributing to excessive neuronal excitability. The combination of a decreased GABA/Glx ratio with an elevated Glx/NAA ratio may enhance the specificity for ^1^H-MRS detection of ALS and ability to monitor glutamatergic and GABAergic targeted therapeutics. Additional longitudinal studies calculating the exact value of these ratios are needed to test these hypotheses and understand how ratios may change over the course of disease progression. Proposed modifications to the experimental design of the reviewed ^1^H MRS studies may also increase the sensitivity of the technology to changes in these neurochemicals, particularly in early stages of disease progression.

## Introduction

Amyotrophic lateral sclerosis (ALS) is a neurodegenerative disease characterized by progressive muscle atrophy as a result of upper and lower motor neuron death. Common symptoms include muscle weakness and stiffness, slurred speech, and difficulty swallowing and breathing. The heterogeneous presentation of symptoms among patients, lack of definitive biomarkers and diagnostic tests, and multitude of differential diagnoses make ALS difficult to diagnose, with the time between symptom onset and diagnosis often exceeding 12 months (1). At present, there is no effective treatment or cure for ALS, with the median survival time around 2–4 years following diagnosis (2). The limited therapies that are available, however, are able to extend life expectancy by around 3–6 months, with better prognoses resulting when therapies are begun sooner. Therefore, there is a need to develop effective biomarkers in order to diagnose ALS earlier and monitor disease progression.

By definition, a biomarker is a medical sign in the body that can be “measured accurately and reproducibly” and that can “influence or predict the incidence of outcome or disease” (3). In general, in order to monitor disease progression over time, biomarkers sensitive to the physiological changes that occur in ALS are needed, but these biomarkers don't necessarily need to be specific to ALS. In order to diagnose ALS, however, biomarkers specific to ALS are needed. Other than clinical measures, there are currently neither specific nor non-specific biomarkers for ALS (4). This lack of biomarkers makes diagnosing individuals and monitoring disease progression challenging. Identification of reliable biomarkers would allow for faster diagnoses and sooner initiation of therapies. In addition, reliable biomarkers could lead to substantial improvements in the ability to assess the efficacy of drugs in clinical trials.

Common biomarkers are often proteins or other molecules in blood, urine, and cerebrospinal fluid. To date, there has been limited success in finding reliable biomarkers in these samples with high enough sensitivity and specificity to diagnosis and monitor disease progression in ALS patients. In addition, MRI and CT scans can be used to identify structural changes in anatomical structures, but because ALS is characterized by motor neuron death rather than significant structural changes, the application of these tests is only helpful when excluding a diagnosis of ALS. Studies using PET imaging to quantify neuroinflammation in the brain have revealed significant differences between healthy controls and ALS patients, but data are preliminary and sample sizes have been small, leading authors to conclude that caution should be taken against “over-interpretation” of findings (4–6). Nevertheless, neuroinflammation is not specific to ALS, so while its detection *via* PET imaging may eventually be a tool that could be used to monitor disease progression, it could not be used as a diagnostic measure.

In this review we focus on recent applications to study ALS through a form of MRI referred to as magnetic resonance spectroscopy (MRS). MRS differs from the more commonly used MRI methods because it provides measurements and images of tissue neurochemical composition. This unique property gives it the potential to reveal biomarkers that are *specific* to ALS and *sensitive* enough to diagnose patients at early stages in disease progression, before symptoms are largely noticeable. We focus on ^1^H MRS, which detects the signal from the MRS active ^1^H nucleus, which is also used in MRI, and in particular the use of MRS to measure the neurochemicals glutamate, GABA, and glutamine. The levels of these neurochemicals have been shown to provide information on mitochondrial energetics and the major excitatory and inhibitory neurotransmitter levels and fluxes (glutamate and GABA, respectively), but limited reviews exist of results observed across studies in ALS patients. Because there have been several excellent reviews on changes in choline, creatine and NAA in MS we do not cover the changes in these compounds here other than as relevant to interpreting the glutamate, glutamine and GABA measurements (7, 8).

In section A Model for Changes in MRS Measured Glutamate, GABA, and Glutamine Levels During the Progression of ALS we present a summary of current theories of how mitochondrial energetics and an altered glutamate/GABA neurotransmitter flux balance play a role in the pathogenesis of ALS. The theories are synthesized into a model that predicts how pathogenesis impacts glutamate, GABA, and glutamine concentrations. The predictions are then compared in section Review of ^1^H MRS Studies Evaluating Glutamate or Glx, GABA, and Glutathione in ALS with the results of all of the MRS studies of ALS to date that have measured one or more of these neurochemicals.

Although there have been a limited number of studies, we found that when normalized for neuronal volume using the MRS biomarker N-acetyl aspartate, there is clear evidence for an elevation of glutamate in the early progression of ALS which is consistent with the predictions of the model of increased activity of glutamatergic neurons leading to excitotoxicity. In addition, several findings of a decrease in GABA levels is consistent with a loss of inhibitory regulation contributing to excessive neuronal excitability. In section Discussion we make recommendations for how ^1^H MRS studies of ALS can be made more sensitive to changes in these neurochemicals. We also address several treatment strategies for ALS targeted at both neurons and glia and assesses whether their mechanisms are amenable for use of MRS as a treatment biomarker.

## A Model for Changes in MRS Measured Glutamate, GABA, and Glutamine Levels During the Progression of ALS

The purpose of this section is to present a model for relating mechanisms of pathogenesis in ALS to fluxes and concentrations quantifiable by MRS. The model is based upon present hypotheses regarding the pathogenesis of ALS, with a primary focus on initial excessive glutamate release followed by a failure of astrocytic glutamate uptake leading to elevated extracellular glutamate levels. These levels lead to excitotoxicity, mitochondrial damage with resultant increases in reactive oxygen species (ROS) and as a consequence apoptosis and a loss of synaptic and neuronal volume. Similar models have been presented previously including for ALS (2) and depression and stress related disorders (9). However, our presentation differs by focusing on the changes in the MRS measured levels of glutamate, GABA, and glutamine due both to changes in their intracellular concentrations and loss of neuronal and tissue volume.

### Increased Neuronal Excitability and Its Impact on Mitochondrial Energy Production, the Glutamate/Glutamine Neuronal Cycle, and Cellular Concentrations of Glutamate and Glutamine Early in the Progression of ALS

We describe here the anticipated effects of progression of ALS on the concentrations of glutamate and glutamine measured by MRS. As later described in section Review of ^1^H MRS Studies Evaluating Glutamate or Glx, GABA, and Glutathione in ALS, the majority of MRS studies performed to date in ALS patients are at 1.5 and 3.0 T, which frequently report the combined signal of glutamate and glutamine, defined as Glx. In the description below, we focus on the level of glutamate, which is the main component of the Glx signal, but acknowledge that we focus on Glx in section Review of ^1^H MRS Studies Evaluating Glutamate or Glx, GABA, and Glutathione in ALS when describing ^1^H-MRS studies performed to date in ALS patients. That said, all predictions about ^1^H-MRS reported glutamate in this section are the same as what would be predicted for ^1^H-MRS reported Glx.

**Figure 1:**
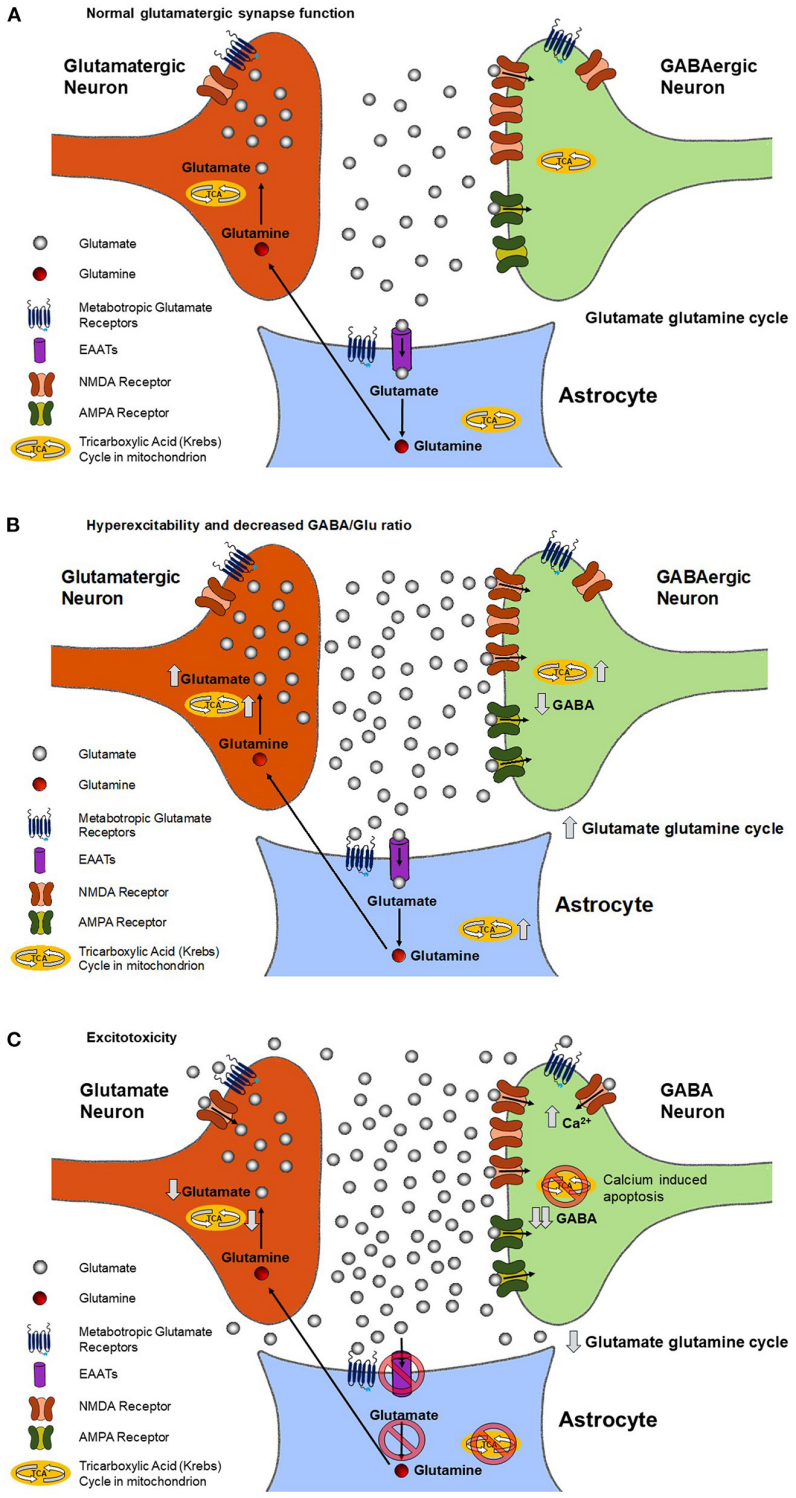
Predicted changes in the rate of mitochondrial ATP production, the rate of the glutamate/glutamine neurotransmitter cycle, and extracellular and intracellular glutamate and GABA concentrations during the progression of ALS in a glutamatergic synapse. During normal neuronal signaling **(A)**, the glutamate-glutamine cycle keeps the synaptic and extracellular concentration of glutamate in a state of homeostasis. As described in the text, in the cycle glutamate released by the neuron, after binding receptors on the post synaptic terminal, is taken up by glial glutamate transporters. Once in the glia glutamate is converted to glutamine by the action of glutamine synthestase. Glutamine is the transported out of the glia and taken back up in the neuron, where it is converted back to glutamate by phosphate activated glutaminase to complete the cycle. During chronically elevated neuronal signaling **(B)**, such as is in ALS, there is an increase in the rate of glutamate release and the glutamate-glutamine cycle rate. The increase may overwhelm the ability of the glia to remove glutamate from the synapse, resulting in increased extracellular glutamate, which further increases neuronal excitability. The increase in the glutamate/glutamine cycle, and resultant release of K+ due to neuronal depolarization, requires additional energy to be spent in the form of ATP by both neurons and glial cells. The large majority of this ATP need is met by increasing the rate of their TCA cycles. The increase in the TCA cycle and glutamate/glutamine cycle subsequently leads to an increase in intracellular glutamate. Chronically elevated neuronal excitability **(C)** causes glutamate to bind synaptic and extrasynaptic NMDA receptors, resulting in increased intracellular Ca^2+^. Increases in intracellular Ca${}_{2}^{+}$ leads to apoptosis of neurons and accompanying reduction of neuronal density characteristic of advanced stages of the disease. As described in Figure 2 the reduction in neuronal density will lead to a decrease in the ^1^H MRS measured Glx, GABA and NAA levels. However, our review of the literatures supports that normalization *via* the NAA signal intensity with the Glx/NAA may be sufficient to allow the predicted neuronal increase in glutamate to be detected.

Glutamate is the primary excitatory neurotransmitter in the human brain and is released by a class of neurons referred to as glutamatergic. Figure 1A shows a schematic of what is referred to as an excitatory synapse that shows the glutamate/glutamine cycle pathway (10, 11). The presynaptic terminal which releases the neurotransmitters is from a glutamatergic neuron and the dendrite it synapses on is from an inhibitory interneuron that synthesizes and releases GABA (referred to as GABAergic). The synapse is surrounded by membranes from peri-synaptic processes of astrocytes. When the presynaptic terminal depolarizes due to an action potential it has a probability of releasing a vesicle containing glutamate. Once released glutamate binds receptors mainly on the post synaptic terminal resulting in the terminal depolarizing. The released glutamate then binds primarily astrocyte glutamate transporters (excitatory amino acid transporters: EAATs), which are present at high density on astrocytic end processes (12), and then are transported into the astrocyte Once in the astrocyte glutamate is rapidly converted by the enzyme glutamate synthase to glutamine, which is a neutral amino acid that has no effect on neuronal excitability. The glutamine is then released from the astrocyte and taken up by glutamatergic (and GABAergic) neurons. In the glutamatergic neuron, glutamine is converted back to glutamate by the action of the enzyme phosphate activated glutaminase (PAG) (13).

Figure 1B shows the effect of increased neuronal excitability on metabolite and neurotransmitter fluxes and levels. Higher neuronal excitability refers to an increase in the probability of glutamate neurotransmitter release for the same signaling input to the synapse. In ALS and other neurological disorders such as epilepsy, a key component of increased excitability is believed to be a decrease in the release or receptor strength of the inhibitory neurotransmitter GABA. As a result, less excitatory input is needed for a neuron to depolarize and release neurotransmitters. Increased excitability also increases the glutamate/glutamine neurotransmitter cycle flux as well as release of K^+^ associated with pre and post synaptic depolarization and uptake of K^+^ by the astrocytes. To restore ionic and glutamate homeostasis requires an increase in mitochondrial ATP production in the pre-synaptic and post synaptic neurons as well as the astrocytes. The increased excitability increases the glutamate/glutamine neurotransmitter cycle flux as well as release of K^+^ associated with pre and post synaptic depolarization and uptake of K^+^ by the astrocytes. To restore ionic and glutamate homeostasis requires an increase in mitochondrial ATP production in the pre-synaptic and post synaptic neurons as well as the astrocytes in order to fuel their Na^+^K^+^ ATPases. The increase in mitochondrial energy production, which is coupled to the glutamate pool *via* exchange with alpha ketoglutarate in the mitochondria, and the glutamate glutamine neurotransmitter cycle leads to an increase in neuronal glutamate concentration (13–15). This increase in glutamate concentration potentially further increases the amount of glutamate released.

### Excitotoxic and ROS Damage During Progression of ALS and Impact on Mitochondrial Energy Production, the Glutamate/Glutamine Neurotransmitter Cycle, and Cellular Concentrations of Glutamate and Glutamine

**Figure 2:**
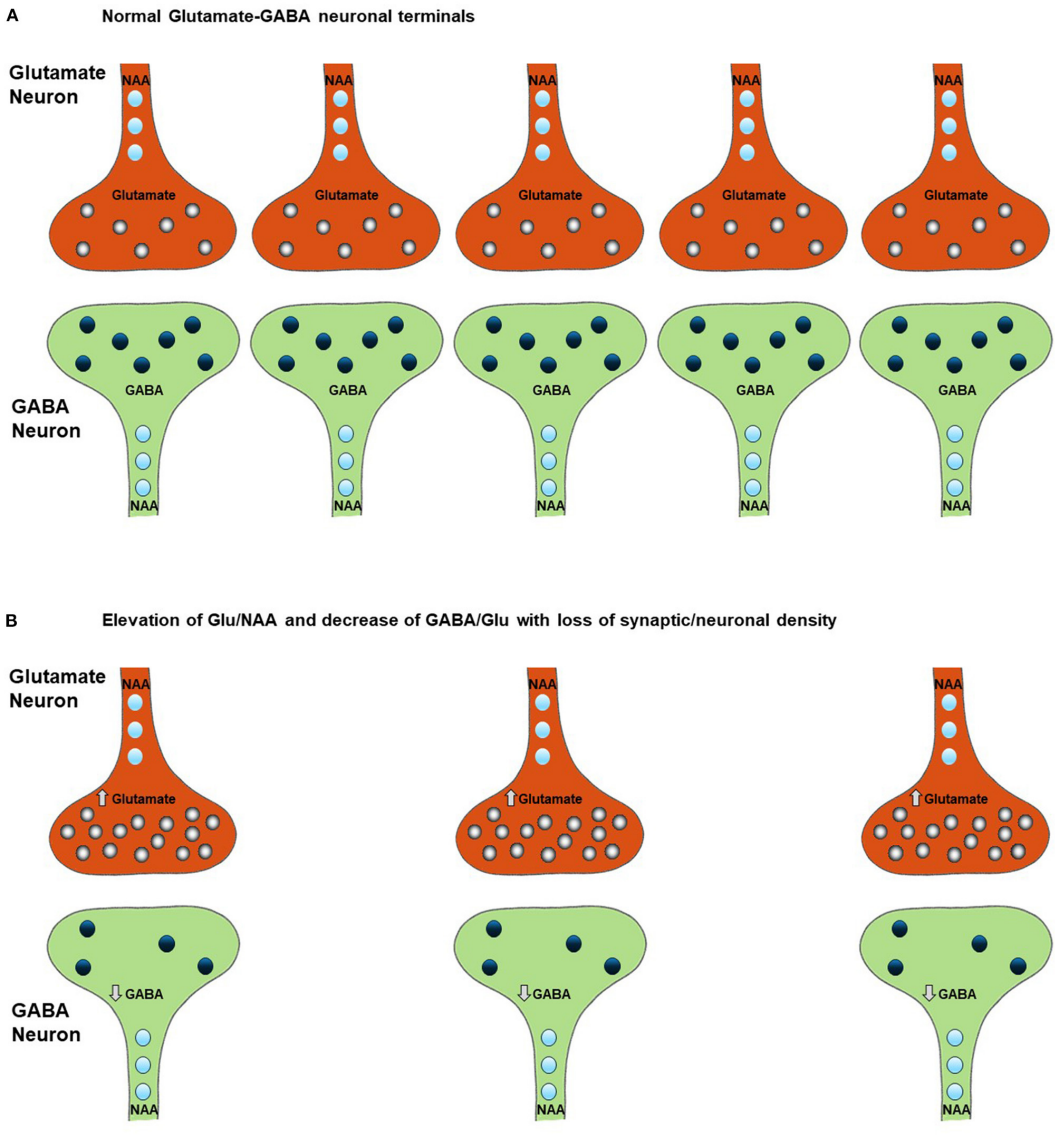
Effect of neuronal and synaptic loss on MRS measured levels of Glx, glutamate, GABA, and NAA. Chronically elevated neuronal excitability leads to a decrease in neuronal density **(B)** relative to normal conditions **(A)** as a result of glutamate excitotoxicity leading to Ca^2+^ induced apoptosis. This excitotoxicity is triggered by an increase in intracellular glutamate, as shown in Figure 1C, and is accompanied (and potentially preceded) by a decrease in intracellular GABA, as described in section Potential Alterations in the GABA and Glutamate Balance in ALS and Impact on Cellular GABA Concentration. Comparison of figures **(A,B)** shows that the increase in glutamate and decrease in GABA due to disease progression may not be detected due to the reduction in their ^1^H-MRS measured levels as a consequence of loss of neurons However the changes due to ALS within the neurons of the ratios of Glu/NAA and GABA/Glu will not be effected by the drop in neuronal density. Therefore, these ratios may be more sensitive to the specific neuronal changes associated with the progression of the disease, which the results of our review of the literature support (see section A Model for Changes in MRS Measured Glutamate, GABA, and Glutamine Levels During the Progression of ALS for a more detailed explanation of the impact of a change in neuronal density on the ^1^H MRS measurements).

Figure 1C shows the excitotoxic effect of chronic elevated neuronal excitability and glutamate release. A combination of increased rates of neuronal glutamate release and reduced glial uptake of glutamate (see below) result in glutamate diffusing out of the synaptic cleft and binding NMDA and metabotropic glutamate receptors outside of the synapse, a process referred to as synaptic spillover. The binding to metabotropic glutamate receptors is believed to act as a safety mechanism that reduces neuronal excitability. However, the increased binding of NMDA receptors leads to increased post synaptic Ca^+^ uptake and resultant excitotoxic loss of synapses and neurons *via* apoptotic mechanisms (16, 17), as shown in Figure 2B.

In addition to direct glutamate excitotoxic mechanisms, additional cellular damage can accumulate *via* ROS production. Excessive mitochondrial activity, and in particular large swings from high to low mitochondrial activity as would happen after a burst of glutamate release followed by inhibition by extra synaptic metabotropic receptors (Figures 1B,C), has been shown to lead to the largest amount of ROS production by mitochondria (16). Excess ROS can inactivate EAAT2 glutamate transporters in astrocytes, reducing the clearance of glutamate (18) and further increasing glutamate spillover. Further impairment of glial glutamate uptake may occur due to a reduction in the enzyme glutamine synthetase that converts glial glutamate to glutamine, as has been shown for epilepsy (19). This excess glutamate leads to additional increases in neuronal intracellular Ca^2+^, which impairs mitochondrial energetics and initiates cytoskeletal degradation. Buildup of excess intracellular Ca^2+^ causes the dendrites to retract and leads to activation of hydrolytic enzymes, initiation of cytoskeletal degradation, and eventual apoptosis (20). Increased intracellular Ca^2+^ can also enter the mitochondria and lead to increased mitochondrial dysfunction, a vicious cycle of mitochondrial damage, oxidative stress, and glutamate-mediated excitotoxicity, and apoptosis of motor neurons (Figure 2B), a hallmark of ALS (21).

The reduction in astrocytic glutamate uptake in combination with reduced mitochondrial energy production is anticipated to result in a decrease in the rate of the glutamate glutamine neurotransmitter cycle and potentially neuronal glutamate concentration. In animal models of chronic unpredictable stress, a condition that due to excessive neuronal stimulation and resultant glutamate release also leads to excitotoxic damage and synaptic loss, it has been shown that there is initially an increase in extracellular glutamate levels, and later a reduction in the glutamate/glutamine neurotransmitter cycle, and mitochondrial energy production (9, 22). Similarly reduced rates of mitochondrial energy production and the glutamate/glutamine neurotransmitter cycle have been observed in chronic human epilepsy associated with mesial temporal sclerosis, a condition characterized by excitotoxic damage and y elevated extracellular glutamate despite hypometabolism during the interictal period, which is associated with a decrease in the concentration of glutamate (23).

### Potential Alterations in the GABA and Glutamate Balance in ALS and Impact on Cellular GABA Concentration

The balance between GABA and glutamate neurotransmission is essential to normal brain function and is believed to be impaired in ALS (2). Once released from interneurons, GABA binds to receptors on the cell body of motor neurons, causing an influx of Cl^−^ and K^+^, which returns the membrane potential back to resting state (24). The relative rates and timing of glutamate and GABA neurotransmitter release and associated membrane depolarization are critical for motor circuit function. In addition to mechanisms associated with neuronal signaling, GABA can also influence neuronal excitability due to neuronal transporter reversal releasing GABA from neurons into the synaptic and extra-synaptic space. In patients with epilepsy, reduced cortical GABA concentration has been shown to be associated with increased seizure frequency (25). Increased cortical excitability has also been associated with reduced GABA concentration in a range of other neurological disorders as well as in cortical plasticity associated with learning (26). Based on these findings, reduced cellular GABA concentration is predicted to be associated with hyperexcitability in ALS (Figures 1B,C). As shown in Figure 2, reduced cellar GABA is associated with decreased synaptic/neuronal density of GABAergic and glutamatergic neurons.

With the progression of ALS there may be an additional decrease in GABA concentration due to a decrease in glutamine released from the glutamate/glutamine neurotransmitter cycle being required for net GABA synthesis (11). It is also noted that GABA synthesis is dependent on intermediaries from the TCA cycle. As such, deficits in mitochondrial energetics that are found in ALS can also lead to reduced production of GABA (27). Figure 1C shows the additional decrease in cellular GABA predicted with further progression of ALS.

### Mitochondrial Dysfunction in ALS and Its Potential Impact on Glutamate, GABA, and Glutamine Concentrations

The details of the molecular mechanisms that lead to disease in ALS are not fully understood. However, one of the earliest pathophysiological events that occurs in ALS may be mitochondrial dysfunction (28), potentially triggered by excessive excitatory glutamate release and ROS production. The mitochondria produce ATP, the molecule that stores and transports energy to cells, through the tricarboxylic acid (TCA) cycle and electron transport chain (ETC). In ALS, there is decreased activity in both of these pathways (28, 29). Decreased ATP production in ALS is linked to mutations in mitochondrial proteins and disruptions to mitochondrial structures and networks (28). In addition, in the case of the SOD1 mutation, the most common of the identified genetic mutations associated with human ALS, misfolded SOD1 proteins in the mitochondrial matrix form aggregates that reduce the permeability of the membrane to ADP, thereby reducing the production of ATP in the electron transport chain (29).

The association of ALS with mutations that impact mitochondrial function however does not explain by itself why symptoms usually begin late in adulthood. A contributing factor may be that mutations in mitochondrial function make the mitochondria more susceptible to damage during periods of elevated glutamatergic neuronal signaling associated with hyperexcitability. Over time as synapses are lost the remaining synapses need to be driven at higher firing rates to compensate, further accelerating the process of damage and cell loss. If this explanation is valid, the same sequence of events shown in Figure 1 is expected with mutations or other factors that reduce mitochondrial energy production (or increase ROS production) making the neurons and glia more susceptible to excitotoxicity. Low levels of GABA would not necessarily be predicted however until the later stages of the disease (Figure 1C), unless they contribute to the initial development of glutamate hyperexcitability.

### Overview of MRS and Measurement of Glutamate, GABA, and Glutamine in the Presence of Neuronal Loss

Magnetic resonance spectroscopy (MRS) is a method that uses the same physical principles as Magnetic Resonance Imaging (MRI) and the same medical equipment. Although even early MRI systems were capable of performing MRS, the number of research and clinical scans using MRS has been considerably lower, largely due to the lower sensitivity and therefore greater technical demands of the technology. Both methods depend on having high field magnets in which magnetic resonance sensitive nuclei have their spin property polarize parallel to the static magnetic field. By applying a radio frequency excitation, the nuclei spins will resonate and emit a radio frequency signal that is detected by the MRI scanner.

Where MRS differs from MRI is both in determining the location of the signal, which is the basis of structural MRI, and in measuring the frequencies of the signals coming from nuclei in different chemical bonds. These frequencies are characteristic of the neurochemicals they arise from and allow maps to be made of the distributions of important neurochemicals such as glutamate, GABA, and many others.

At 7.0 T the best case spatial resolution is from magnetic resonance spectroscopic imaging (MRSI) measurements of glutamate and glutamine for regions deep within the brain such as the hippocampus is ~0.5 cm^3^ (30). Approximately 3–4-fold better volume resolution can be obtained for regions of the cerebral cortex near the surface of the brain (30). Single volume MRS measurements of glutamate are usually obtained at lower spatial resolution than MRSI. For GABA the achievable spatial resolution is ~10-fold lower than for glutamate due to its lower concentration (~5 cm^3^). At 3.0 T the spatial resolution for glutamate, glutamine and GABA are ~2–2.5 times lower due to sensitivity increasing approximately linear with field strength. Even for the highest spatial resolution measurements, the MRS/MRSI signal is the sum of the MRS signal from many thousands to millions of neurons and astrocytes (note: extracellular concentrations of glutamate and GABA are several orders of magnitude lower than intracellular concentrations and can be neglected). MRS is a linear method so that the total signal is proportional to the total number of molecules within the measurement volume after appropriate correction factors are applied. Using glutamate as an example, and assuming for simplicity that the concentration of glutamate is the same in all neurons, the MRS signal from neuronal glutamate is given by:

$$\begin{array}{l} {\left(\text{Intensity Glutamate Signal}\right)}_{\text{MRS}}\;\sim \;\left(1/voxel\;volume\right)\;*\\ \left({\left[\text{Glu}\right]}_{\text{neuron}}\;*\;fractional\;volume\;neurons\right) \end{array}$$

As seen in the equation, if the fractional volume of the neurons in the voxel decreases due to synaptic loss (or total neuron number loss), the MRS glutamate signal will decrease proportionately. As a consequence a decrease in neuronal fractional volume may cancel out the impact of an increase in intracellular glutamate concentration predicted during the early progression of ALS, as illustrated in Figure 2. However, as shown in epilepsy and other disorders (23), the impact of a loss in neuronal volume can be at least partially corrected for by dividing the signal intensity from glutamate by the intensity of the signal from the neurochemical N-acetyl aspartate (NAA). N-acetyl aspartate is synthesized by neuronal mitochondria and is only found in measurable (by MRS) amounts in neurons. Under conditions of neuronal or synaptic loss its intracellular concentration either stays the same or decreases. Even if a loss of synaptic density reduces the total signal from glutamate, an increase in the ratio of the signal from glutamate to the signal from NAA as predicted during the progression of ALS will still be detectable. In section Review of ^1^H MRS Studies Evaluating Glutamate or Glx, GABA, and Glutathione in ALS we retrospectively apply this approach to the meta –analysis of ^1^H MRS studies of ALS to test the predictions of the model, which are summarized in Table 1.

**Table 1:** Summary of predicted MRS detectable changes in the pathogenesis of ALS.

**Ratio**	**Predicted MRS detectable change in ALS**
[Glutamate]/[NAA][Glutamine]/[NAA][Glx]/[NAA]	Increase
[GABA]/[Glutamate][GABA]/[Glutamine][GABA]/[Glx]	Decrease

*MRS detects the overall number of molecules within a fixed volume, and therefore loss of glutamatergic and GABAergic neurons may cancel out observed changes in neurotransmitter concentration on a per-neuron basis. Given that NAA is found only in neurons and its MRS signal intensity is correlated with neuronal density, ratios of overall signal intensity of glutamate, glutamine, Glx, and GABA to overall signal intensity of NAA can provide an estimate for the per-neuron concentration of each neurotransmitter. In the pathogenesis of ALS we would expect the concentration of glutamate, glutamine, and Glx per neuron to increase; the concentration of GABA per neuron to decrease; and the overall concentration of NAA to decrease. Ratio predictions for are summarized based on this logic. It is noted that predictions listed hold for all stages of the disease. However, in the late stages, extensive neuronal damage and loss of volume may obscure the ability to detect elevated glutamate in the remaining functional neurons*.

### Factors That Determine the Accuracy and Spatial Resolution of the ^1^H MRS Measurement of Glutamate, Glutamine, GABA, and NAA

**Figure 3:**
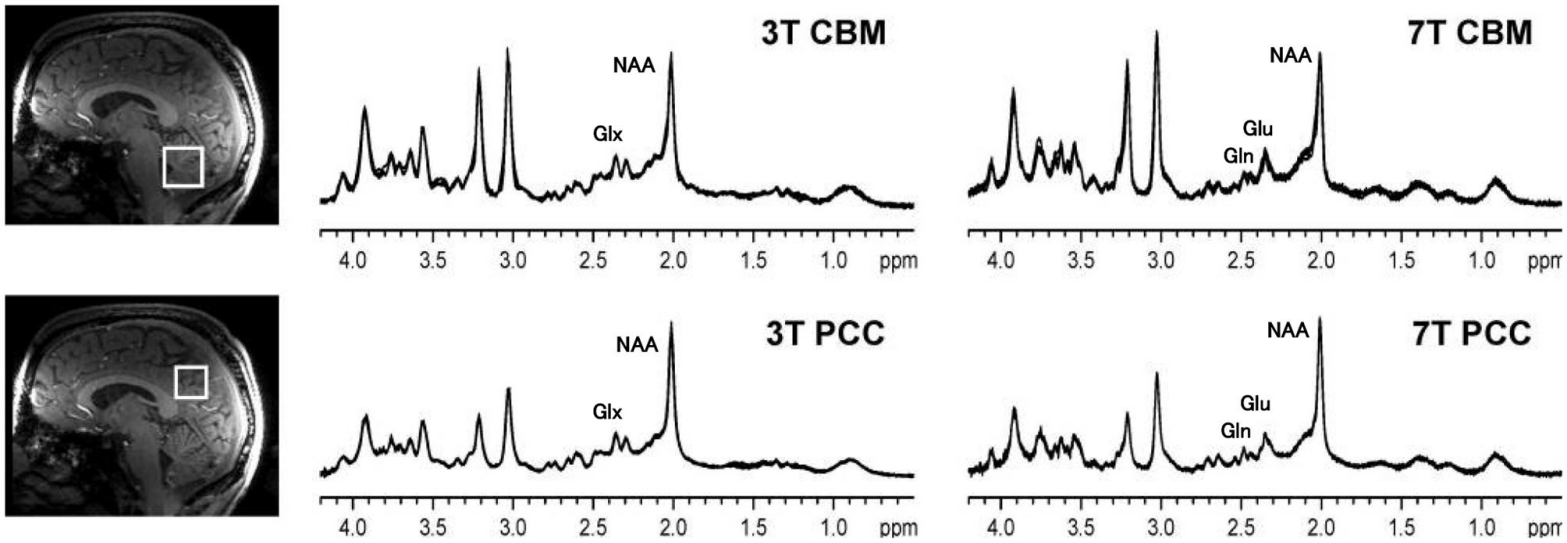
^1^H MRS spectra from the cerebellar vermis (CBM) and posterior cingulate cortex (PCC) of a healthy control sequenced at 3 and at 7 T. Compared to 3, at 7 T the spatial resolution is improved and there is a better signal to noise ratio. At 3 T the resonances of glutamate and glutamine overlap, and therefore Glx is reported, but glutamate and glutamine contribute to both of the peaks near 2.35 ppm. At 7 T, the glutamate and glutamate resonances are cleanly separated. Thus, at 7 T, the signal area of glutamate can be more accurately calculated. The figure is adapted from Figure 1 from Terpstra (35) with permission.

**Figure 4:**
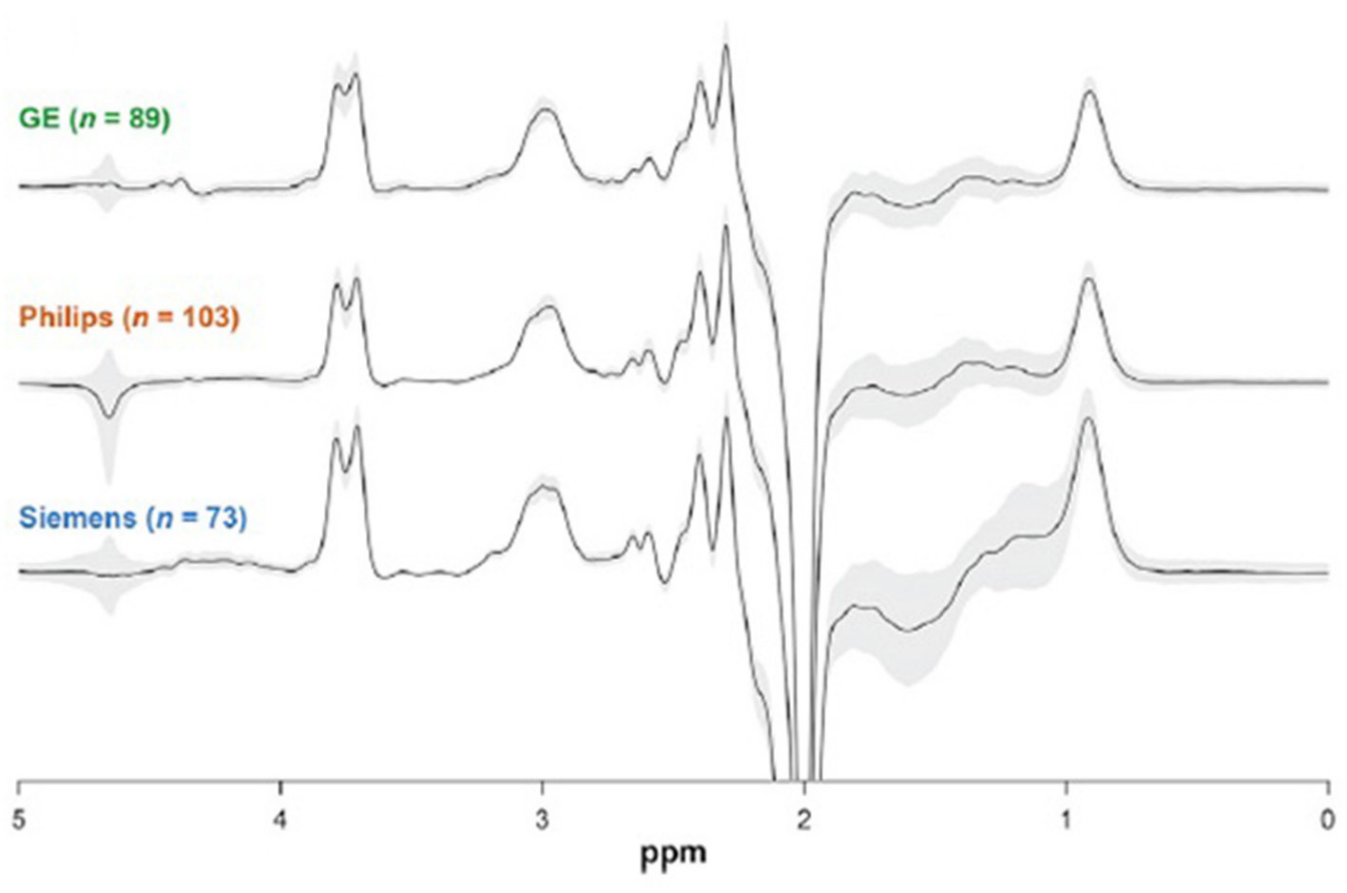
3.0 T GABA J edited spectra of controls obtained at 24 different sites using MRI systems from 3 vendors. The similarity between the spectra explains the high reproducibility found between sites, with a 12% coefficient of variation. Each spectrum shown is the overlay of all the spectra obtained from a vendor, and demonstrates the small coefficient of variation. This figure is adapted from Figure 2 from Mikkelsen et al. (36) with permission.

There have been many excellent books and review articles on MRS and therefore we will not provide a detailed description of the technology here but instead refer to several informative references including recent consensus articles (31–34). However, we briefly present several key factors that influence the MRS measurement accuracy and spatial resolution. We also provide an example of MRS spectra sequenced at different field strengths for glutamate (Figure 3) and using clinical MRI systems from different vendors for GABA at 3 T (Figure 4) at to illustrate these points. In sections Review of ^1^H MRS Studies Evaluating Glutamate or Glx, GABA, and Glutathione in ALS and Discussion we use these factors to propose explanations for some of the differences between early results obtained at a magnetic field strength of 1.5 Tesla (T) and more recent results at 3.0 Tesla (T) and 7.0 Tesla (T). Although there are increasing number of studies that use MRS imaging, known as MRSI, we could not find any that examined glutamate, glutamine or GABA in ALS. Therefore, we focus on single volume MRS studies in our methodological description here and the studies reviewed. We refer interested readers to an excellent recent consensus article on MRSI technology and its applications (37).

The ability to separate peaks is critical for accurate measurement of neurochemical concentrations. The frequency separation between peaks (referred to as resonances) is proportional to the magnetic field strength and therefore is 2.33 times greater at 7 T than 3.0 T. In addition to magnetic field strength, another important factor in spectral resolution is the homogeneity of the magnetic (Bo) field. If the field is not uniform it will broaden the peaks and therefore increase their overlap, making accurate measurement more difficult.

To illustrate the spectral resolution advantage of 7.0 T, Figure 3 shows an example of ^1^H MRS spectra obtained from control subjects, using the same pulse sequence, obtained at a magnetic field strength of 3.0 and 7.0 T. The number of peaks that can be identified in the spectra is greater at 7.0 T than at 3.0 T, as shown by the labels assigning each peak to a neurochemical. At 3.0 T the peaks of glutamate and glutamine are overlapped and only the combined measurement, referred to as Glx, can be performed. Because the dominant component of the Glx combined resonance is glutamate, the hypotheses presented here about the time evolution of glutamate in ALS also applies. In section Review of ^1^H MRS Studies Evaluating Glutamate or Glx, GABA, and Glutathione in ALS we therefore also include studies that reported only Glx. Although potentially higher precision for measuring Glx (and Glu directly) is available at 7.0 T, as described in recent consensus papers the present coefficient of variation of the NAA and Glx measurements at 3.0 T, normalized to either creatine or water, is on the order of 10% or less between control subjects with even better precision in repeat scans on individual subjects (31–34).

In MRS the strength of the radiofrequency signal emitted from each neurochemical is proportional to its concentration. However, MRS is a spectroscopic method and therefore needs to be calibrated in order to convert the measurement to neurochemical concentration. There are two main methods for performing this calibration. The most common is internal referencing in which the intensity of the peak from the neurochemical of interest, such as glutamate, is compared with the intensity of a peak from a neurochemical that is known to be stable in concentration, such as creatine, or alternatively NAA or water. After correction for differences in the efficiency of detection, the concentration can be assigned. External referencing is when the signal obtained from a volume in the brain is compared with the signal obtained with the same MRI system at the same position from a large sphere or other container containing a solution of the same compound of known concentration. There are advantages and disadvantages of both methods which have been reviewed in the literature (34). Of particular relevance for MRS of ALS is that the tissue composition of effected brain regions will change with disease progression and with loss of neuronal volume. In our review of MRS measurements of glutamate in ALS, we suggest that the internal reference be changed to NAA to compensate for that loss and obtain a more accurate estimate of changes in glutamate concentration at the neuronal level.

Once the internal or external calibration is performed, the spectra obtained need to be fitted in order to measure the intensity of each peak. There are a variety of methods that are used to perform peak fitting and we refer the interested reader to two recent excellent reviews (34, 38). The peak fitting program most commonly used is called LC Model (39). The program fits MRS spectra as a linear sum of the resonances (often referred to as “peaks”) from each neurochemical present in the measured volume. The ability of LC Model to accurately measure peak intensity depends on the sensitivity of the measurement and how well the peaks are separated. If the peaks are poorly separated, as can occur at lower magnetic fields, often it is used to fit the combination of the two peaks instead such as for the combined peak of glutamate and glutamine (Glx). In addition, LC model is less accurate if it needs to separate peaks from neurochemicals from broad overlapping peaks from macromolecules (e.g., soluble proteins and lipids) and partially suppressed water (40). However, there are spectroscopic methods for suppressing these broad peaks that can improve the neurochemical peak fitting accuracy as reviewed in the following references (34, 41, 42).

In some cases, even at high magnetic fields it is not possible to measure peaks from low concentration neurochemicals such as GABA and glutathione due to overlap with the 10-fold or more higher peaks of creatine, NAA, and other metabolites. In order to allow their quantitation, specialized methods for acquiring the MRS signal, referred to as spectral editing methods, have been developed. These methods take advantage of the unique MRS properties of individual neurochemicals to obtain spectra which contain only their peaks, greatly simplifying accurate measurement. Several references that review the methodological requirements for spectral editing are provided here (43–46). In addition, Figure 4 shows a comparison between GABA edited spectra at 3 T from 24 different sites using three different MRI vendors (36). Despite the multiple sites and different vendors an average coefficient of variation of 12% was found, with lower values at individual sites. This result indicates that with state of the art spectral editing there is sufficient precision to pick up significant reductions or increases in GABA levels.

For *in vivo* MRS measurements the sensitivity of the measurement is proportional to the field strength and the concentration of the neurochemical being measured. Sensitivity is also inversely proportional to the volume resolution of the measurement. For example, a 10 min MRS measurement can be obtained at 4.66-fold better volume resolution at 7 vs. 1.5 T. The higher sensitivity for MRS, and MRI, is a major factor for the increasing use of higher field MRI magnets. In addition to the magnetic field strength the sensitivity depends upon how efficient the radiofrequency receiver coils that are used. Over the last 15 years due to the implementation of what is referred to as phased array technology, this sensitivity has increased by a factor of 3–4 times. Therefore, a modern MRS measurement at 7 T can be obtained from volumes as much as 16 times smaller than a measurement performed in the 1990s at 1.5 T. This increased volume resolution allows MRS studies of functionally specialized regions of the brain and regions outside of the brain such as the brain stem and even the spinal cord.

## Review of ^1^H MRS Studies Evaluating Glutamate or Glx, GABA, and Glutathione in ALS

The purpose of this section is to provide an overview of published studies that have used ^1^H MRS to identify possible molecular biomarkers for ALS. Our focus is on the neurotransmitter amino acids glutamate (or Glx) and GABA. We also discuss changes in NAA, which have been reviewed previously due to its relevance for distinguishing neuronal volume loss from changes in cellular glutamate and GABA levels.

We will attempt to interpret the changes observed in each molecule from the standpoint of whether or not they support the predictions of the model in section A Model for Changes in MRS Measured Glutamate, GABA, and Glutamine Levels During the Progression of ALS. Among the caveats to the interpretation of the present studies is that the number of studies and patients per study have been limited, and particularly for the earlier studies there have been methodological limitations in the accuracy of the measurements. Despite these and other limitations, when evaluated as a group there were several promising trends that support the ability to use MRS to potentially improve the differentiation from other neurodegenerative disorders and to follow disease progression and treatment.

In many of the studies discussed, the functional status of patients was evaluated using the ALS Functional Rating Scale (ALSFRS). This questionnaire evaluates a patient's physical status, including measures of bulbar and motor function, as well as their independence and ability to perform daily tasks, such as walking up a flight of stairs or getting themselves dressed. Since ALS has no definitive diagnosis, the ALSFRS evaluation can aid in a diagnosis of suspected, possible, probable, or definite ALS. This evaluation can also be used to qualitatively assess disease progression over time, and often serves as inclusion and exclusion criteria for enrolling patients in studies.

### Methodology

To the best of our knowledge, this is a comprehensive review of all published papers reporting measurements of glutamate (or Glx) and GABA in ALS patients as of November 2020. PubMed was use to do a search of key terms for ALS (ALS, amyotrophic lateral sclerosis, neurodegeneration, motor neuron disease), in combination with key terms for MRS (MRS, proton MRS, magnetic resonance spectroscopy, ^1^H-MRS) and the above metabolites. Studies were then reviewed for study methodology and results. A separate search was done for glutamate and Glx, and for GABA. Studies were excluded if they did not use specifically ^1^H-MRS to measure at least one of the above metabolites, if they did not report original data, and if the patient population was not specific to ALS patients. Most studies did not provide a distinction of sporadic ALS vs. familial ALS.

Although not presented in the papers reviewed, we calculated for each study the Glx/NAA ratio and GABA/Glx ratio, where applicable, in ALS patients and in controls, using the mean values reported for each metabolite. As discussed in section A Model for Changes in MRS Measured Glutamate, GABA, and Glutamine Levels During the Progression of ALS, the spectra of glutamate and glutamine overlap at low field strengths, and results are reported as Glx. Given that most clinical MRI scans operate at 3.0 T at present, and the fact that nine out of thirteen studies reviewed measured Glx, we elected to study Glx for this review. For studies reporting glutamate, rather than Glx, absolute concentration of Glx was calculated by summing the reported values of glutamate and glutamine. It is noted that predictions discussed in section A Model for Changes in MRS Measured Glutamate, GABA, and Glutamine Levels During the Progression of ALS about ^1^H-MRS measured glutamate also apply to ^1^H-MRS measured Glx.

Table 1 describes our predictions for the change in Glx/NAA and GABA/Glx ratios in ALS patients relative to healthy controls, based on the model presented in section Overview of MRS and Measurement of Glutamate, GABA, and Glutamine in the Presence of Neuronal Loss. Tables 2, 3 report the ratio of (Glx/NAA)_ALS_ to (Glx/NAA)_Control_. If this ratio is >1, it suggests that the ratio of Glx/NAA was higher on average in ALS patients relative to healthy controls. Table 4 presents the methodology used for each study. Finally, Table 5 reports the average values and range of values for coefficients of variation for Glx, GABA, and NAA for each study. These values were calculated as the standard deviation divided by the mean for each metabolite.

**Table 2:** Results of ^1^H-MRS studies evaluating Glx and glutamate in ALS patients.

**References**	**Metabolite measured**	**Metabolite levels in ALS patients relative to HCs**	**Metabolite correlation with disease status**	**NAA levels in ALS patients relative to HCs**	**Ratio of (Glx/NAA)_**ALS**_ to (Glx/NAA)_**Control**_**	**Ratio in agreement with hypotheses?**
Block et al. (47)	Glx/PCr	No significant difference	No significant correlation	Significant decrease (*p* <0.005)	1.02	Yes
Pioro et al. (48)	Glx /Cr	Significant increase (*p* = 0.02)	Significant inverse correlation between Glx and bulbar ALSFRS score (*r =* −0.68, *p* = 0.03)	Significant decrease (*p* = 0.005)	1.87	Yes
Bradley et al. (49)	Glx/Cr	No significant difference	*Not reported*	Significant decrease (*p < * 0.05) in motor cortex and brainstem	0.94	No
Bowen et al. (50)	Glx	Significant decrease (*p* = 0.048) in motor cortex	*Not reported*	Decrease that approached significance (*p* = 0.059) in motor cortex	0.92	No
Unrath et al. (51)	Glutamate + Glutamine	No significant difference within ALS patient cohort	*Not reported*	Significant decrease (*p* <0.05) in motor cortex	N/A – *HCs not included in study*	N/A
Han and Ma (52)	Glx/Cr	Significant increase (*p* <0.01)	Negative correlation that reached significance with ALFRS score: motor cortex (*r* = −0.47, *p* = 0.08) and PLIC (*r* = 0.25, *p* = 0.08)	Significantly decreased (*p* <0.01)	1.56	Yes
Van der Graaf et al. (53)	Glx/Cr	No significant difference	No significant correlation	Did not differ significantly	1.13	Yes
Foerster et al. (2)	Glx	No significant difference	Significant correlation between Glx levels and UMN score (*r* = −0.63, *p* <0.001)	Significantly decreased (*p* <0.01)	1.11	Yes
Atassi et al. (54)	Glutamate + Glutamine	Significant decrease (*p* = 0.02)	Significant positive correlation between Glutamate and forced vital capacity (*p* = 0.013)	Significant decrease (*p* = 0.004)	1.06	Yes
Cheong et al. (55)	Glutamate + Glutamine	No significant difference	Significant positive correlation with ALSFRS score (*r =* 0.48, *p* = 0.04)	Significant decrease (*p* = 003)	1.04	Yes
Cheong et al. (56)	Glx	Significant increase (*p* = 0.003)	Significant correlation with ALSFRS score in patients with declining bulbar function (*p* <0.0001), but not in those without declining bulbar function	Significant decrease (*p* = 0.01)	1.06	Yes
Weerasekera et al. (57)	Glx	Significant increase (*p* = 0.025)	Significant correlation with ALSFRS score (*r* = −0.6673, *p* = 0.0066)	Significant decrease (*p* = 0.0014)	1.34	Yes
Blicher et al. (58)	Glutamate/Cr + Glutamine	Significant decrease (*p* = 0.002)	No significant correlation	Significant decrease (*p* = 0.002)	0.95	No

**Table 3:** Results of ^1^H MRS studies evaluating GABA in ALS patients.

**References**	**Metabolite**	**Metabolite levels in ALS patients relative to HCs**	**Metabolite correlation with disease status**	**Glx or glutamate levels in ALS patients relative to HCs**	**Ratio of (GABA/Glx)_**ALS**_ to (GABA/Glx)_**Control**_**
Foerster et al. (59)	GABA	Lower than in HCs (*p* = 0.038) in motor cortex	No significant correlation	*Did not measure glutamate*	*N/A*
Foerster et al. (2)	GABA	Lower than in HCs (*p* <0.01)	Significant correlation (*r* = −0.39, *p* = 0.05)	Glx did not differ significantly	0.80
Atassi et al. (54)	GABA	Did not differ significantly from HCs	No significant correlation	Glutamate was significantly lower in ALS patients (*p* = 0.02)	0.80
Weerasekera et al. (57)	GABA	Did not differ significantly from HCs	No significant correlation	Glx was significantly higher in ALS patients (*p* = 0.003)	0.78
Blicher et al. (58)	GABA/Cr	Did not differ from HCs	No significant correlation	Significantly lower in ALS patients (*p* = 0.002)	1.05

**Table 4:** Methodology overview of all ^1^H-MRS studies evaluated.

**References**	**ALS patient cohort (#, disease status)**	**Average age**	**Methodology**	**Area of the brain studied**	**Voxel size (mm)**	**TE base value (ms)**
Block et al. (47)	*N* = 22 Possible, probable, or definite ALS *N* =11 Suspected ALS	ALS: 50Control: 45	1.5 T, PRESS	Motor cortex	40 ×30 ×25	30
Pioro et al. (48)	*N* = 10 Probable or definite ALS	ALS: 48Control: 42	1.5 T, PRESS	Medulla	10 ×10 ×20	40
Bradleyet al. (49)	*N* = 20 Probable or definite ALS	ALS: 54.9Control: 58.4	1.5 T, STEAM	Motor cortex	*Data not available*	20
Bowen et al. (50)	*N* = 18 Probable or definite ALS	ALS: 54Control: 59	1.5 T, STEAM	Motor cortex	20 ×20 ×20	20
Unrath et al. (51)	*N* = 11 Definite ALS	*Data not available*	1.5 T, PRESS	Motor cortex	20 ×20 ×20	30
Han and Ma (52)	*N* = 15 Probable or definite ALS	ALS: 51Control: 49	3 T, PRESS	Motor cortex	20 ×20 ×20	25 (67 effective)
Van der Graaf et al. (53)	*N* = 12 Probable or definite ALS (limb onset patients)	ALS: 58Control: 57	3 T, PRESS	Motor cortex	13 ×25 ×20	35
Foerster et al. (59)	*N* = 10 Probable ALS	ALS: 61.5Control: 58.6	3 T, MEGA-PRESS	Motor cortex	30 ×20 ×30	68 (TE1 = 15, TE2 = 53)
Foerster et al. (2)	*N* = 29 Probable or definite ALS	ALS: 60Control: 59	3 T, PRESS	Motor cortex	30 ×30 ×20	68
Atassi et al. (54)	*N* = 13 Mean ALSFRS score = 38.4	ALS: 56Control: 52	7 T, STEAM	Motor cortex	20 x 20 x 20	5
Cheong et al. (55)	*N* = 20 Possible, probable, or definite ALS	ALS: 57Control: 57	7 T, semi-LASER	Motor cortex	22 ×22 ×22	26
Cheong et al. (56)	*N* = 10 Possible, probable, definite ALS	ALS: 58Control: 58	7 T, semi-LASER	Motor cortex, pons	22 ×22 ×22	26
Weerasekera et al. (57)	*N* = 20 Mean ALSFRS-R score = 30.2	ALS: 56Control: 57	3 T, PRESS	Motor cortex	20 ×20 ×20	22
Blicher et al. (58)	*N* = 9 Mean ALSFRS-R score = 38	ALS: 65Control: 65	3 T SPECIAL	Motor cortex, occipital lobe	25 ×20 ×20	8.5

**Table 5:** Coefficient of variation for all ^1^H-MRS studies evaluated.

	**ALS**			**Control**		
	**Min**	**Max**	**Average**	**Min**	**Max**	**Average**
Glx or Glutamate	3.72%	28.90%	15.14%	4.41%	33.93%	14.18%
GABA	7.17%	77.78%	34.65%	14.12%	51.56%	28.30%
NAA	2.38%	21.90%	11.76%	2.51%	15.75%	8.60%

### Overview of ^1^H MRS Studies of Glutamate and Glx

Thirteen papers were identified that used ^1^H MRS to measure glutamate or Glx levels in ALS patients. Results are summarized in Table 2, and details of MRS methodology are summarized in Table 4. Across studies, findings of glutamate or Glx levels in ALS patients relative to healthy controls were variable: levels were increased in three studies (48, 52, 57), decreased in three studies (50, 54, 58), and showed no significant change in five studies (2, 47, 49, 53, 55). Two studies quantified change in glutamate levels over time in ALS patients, with one study finding non-significant changes in change over time (51) and one study finding increased levels over time (56).

Varying results across studies may be due to several factors, including stage of disease, pulse sequence, and magnetic field strength methodology, as well as variation within the population. We address these possibilities below. In addition, we evaluate the Glx/NAA ratio in ALS patient populations vs. healthy controls as a way to correct for reductions in measured glutamate levels as a result of neuronal loss, thereby allowing of estimate of glutamate level per volume of neurons. We propose that this metric may be specific to a diagnosis of ALS.

#### Stage of Disease

We would have expected ^1^H-MRS measured glutamate and Glx levels to have risen during the pre-symptomatic and/or early symptomatic phase of the disease as a result of elevated glutamatergic activity, and then decline during the later stage of the disease with loss of neuronal volume as well as mitochondrial dysfunction. Therefore, longitudinal studies are critical for assessing the ability of ^1^H-MRS measurements of glutamate to track the disease. Out of the 13 studies reviewed, only two studies (51, 55) evaluated changes in glutamate levels longitudinally over time. Of these studies, only results by Cheong were significant. When following patients over the course of 12 months, the study found a significant increase in glutamate levels in ALS patients, a finding that is in line with our hypotheses about increasing glutamate levels throughout disease progression, and a non-significant change in glutamate levels in HCs. There was no significant difference in glutamate levels in ALS patients relative to HCs at baseline (55), which could reflect the heterogeneity of symptom presentation. This heterogeneity may have caused differential changes in glutamate levels, despite all patients being diagnosed with probable or definite ALS. This occurrence therefore points to the importance of longitudinal studies with large sample sizes, as cross-sectional studies and studies with small sample sizes may not be accurately representative of disease presentation.

#### Pulse Sequence and Magnetic Field Strength Methodology

One limitation in assessing the ^1^H-MRS glutamate biomarker is the ability to control for the varying MRS methods that were used across the studies assessed, as shown in Table 4. Between 1998 and 2020, technology advances led to large improvements in the sensitivity and overall reliability of the measurement. These advances include higher field strength (which improves sensitivity and spectral resolution), improved pulse sequences, and improved spectral analysis methodology (see section A Model for Changes in MRS Measured Glutamate, GABA, and Glutamine Levels During the Progression of ALS). These factors may have contributed to inconsistencies in findings of glutamate levels across studies. Among the 13 studies reviewed, five used 1.5 T field strength (47–51), five used 3 T field strength (2, 52, 53, 57, 58), and three used 7 T field strength (54–56). In general there is a trend of increasing magnetic field strength over time as higher field magnets became available, but it is noted that both Weerasekera and Blicher used 3 T in 2019 after 7 T had been previously used by Atassi and Cheong. Although 7 T magnets have become more widely available for research, the majority of MRI systems use 3 T for neuroimaging applications at present.

At 1.5 T magnetic field strength, the ability to distinguish metabolites is particularly challenging due to the poorer separation of resonances in the spectra than at 3 or 7 T. This condensed spectrum can lead to overlaps in peaks, which can result in miscalculation of metabolite concentration (60). Most notably, the standard LCModel method of analysis (see section A Model for Changes in MRS Measured Glutamate, GABA, and Glutamine Levels During the Progression of ALS) depends on both separation of metabolites with resonances that overlap as separation of metabolites from broad baseline resonances of macromolecules. Macromolecules can change with pathology (61), and at the lower field strengths there is extensive overlap between Glx and other resonances of interest at the 2.35 PPM C4 glutamate resonance that is normally measured. Therefore, when the basis fit is applied, the Glx signal may be underestimated due to overestimating the contribution from baseline resonances (62). This could potentially explain the lower levels of Glx reported by Bowen et al. in ALS patients compared to HCs.

Furthermore, in an ideal experiment, spectra for a given region would be normalized relative to a reference region that is free of pathology as has been applied to epilepsy and other neurological disorders (63). The advantage of this normalization is that it corrects for individual variation in metabolic rates and concentrations which can be on the order of 20% (64). The reference region would be one that is unaffected by ALS. At present the ideal control region is uncertain due to reported disease involvement in non-motor regions (65, 66). However, in Alzheimer's and other neurodegenerative diseases the visual cortex has been found to be particularly resistant to change, and references measurements from that region would be worth obtaining at least on an exploratory basis to assess if it reduced inter subject variation in the measurement of metabolite changes in the disease affected volume. Out of the 13 papers studied, none mentioned use of a reference region, which could possibly explain inconsistent findings when the small size of the study groups is also taken into account.

#### Can ^1^H MRS of the Glx/NAA Ratio Provide Information on Excitotoxicity?

As described in section A Model for Changes in MRS Measured Glutamate, GABA, and Glutamine Levels During the Progression of ALS, elevated extracellular glutamate due to both enhanced neuronal release and impaired glial reuptake plays a key role in theories of the pathogenesis of ALS. An increase in extracellular glutamate leads to the death of motor neurons, a process called excitotoxicity (16) that is a hallmark of ALS (see Figure 2). A limitation of using MRS to study excitotoxicity is that the concentration of glutamate in the extracellular fluid is on the order of 1,000 times lower than the intracellular concentration. As such, using ^1^H MRS to detect increases in extracellular glutamate can be challenging because ^1^H MRS does not distinguish between intracellular and extracellular metabolites (31, 67). However, due to the reported coupling between neuronal activity and glutamate concentration *via* the glutamate/glutamine cycle (see Figure 2), the intracellular concentration of glutamate is believed to reflect neuronal activity (31, 67). A high level of intracellular glutamate during the early stages of the disease therefore may be reflective of excessive glutamate release which could lead to excitotoxic damage.

A complication of assessing excitability from glutamate levels is that elevation in neuronal activity will occur in parallel with progressive mitochondrial damage and apoptotic cell death, both of which would lower glutamate concentrations. A potential way to distinguish these possibilities is to examine together the changes in both glutamate and NAA. Although NAA can decrease due to both cellular density loss and mitochondrial damage, both situations indicate ongoing neurodegeneration (31, 68). In contrast, even with ongoing neurodegenerative processes, excessive glutamate release on a per-neuron basis, reflected by increased neuronal glutamate concentration, may take place. In all but one paper (53) there was a finding of a significant (*p* < 0.05) or approaching significant decrease in NAA levels in ALS patients compared to HCs. NAA is an amino acid located exclusively in neurons and an indicator of neuronal viability and functional integrity if at normal values. A decrease in NAA is not specific to ALS, as it is also seen in other neurodegenerative diseases such as Alzheimer's disease and epilepsy (31, 68). However, in these and other neurological disorders glutamate and NAA usually drop in parallel (23, 69). The level of NAA is often reported along with a drop in glutamate levels as a positive indicator of neurodegeneration in ALS patients. Depending on the pathology, a drop in NAA could reflect a decrease in cellular density but also independently a reduction in mitochondrial activity due to NAA being synthesized in the mitochondria. Nonetheless, potentially in combination with ultra-high resolution MRI to independently measure tissue loss, these two possibilities could be distinguished improving the ability to use NAA as a neuronal volume marker.

Although not presented in the papers reviewed, we calculated for each study the Glx/NAA ratio in ALS patients relative to controls as a normalization factor. These calculations were based on the reported absolute concentrations of each metabolite. As shown in Table 2, in nine out of the twelve studies reporting both Glx (or glutamate + glutamine) and NAA, this ratio of Glx/NAA in ALS patients vs. healthy controls was >1.0, suggesting an increase in neuronal glutamate levels in ALS patients. In seven out of these nine studies with an elevated Glx/NAA the findings were a result of significant increases (48, 52, 56, 57) in Glx, or non-significant changes (2, 47, 55) in Glx or glutamate, in conjunction with significant decreases in NAA in ALS patients relative to healthy controls. Therefore, it is likely that had the ratio been calculated on individual subjects it would have shown statistical significance in the comparison. In the remaining two studies with a ratio > 1.0 (53, 54), significant changes in both Glx or glutamate and NAA relative to healthy controls were not observed, but this does not rule out the possibility that the changes in Glx/NAA ratio could be statistically significant. The importance of using a normalization for tissue and ideally neuronal volume is emphasized by the finding in all of these studies of decreased NAA levels.

Other studies (49, 50, 58) did not observe a ratio of Glx/NAA in ALS patients vs. healthy controls that was >1.0. The study by Bradley did not find a significant change in either Glx or NAA levels in ALS patients relative to HCs. As such, an elevated Glx/NAA ratio could have been missed due to measurement variation between subjects. Studies by Bowen and Blicher, on the other hand, observed significant or near significant decreases or decreases that approached significance in both Glx and NAA in ALS patients, which is not in line with the hypotheses outlined in section A Model for Changes in MRS Measured Glutamate, GABA, and Glutamine Levels During the Progression of ALS.

### Overview of Studies of GABA

Five papers were identified that used ^1^H MRS to measure GABA levels in ALS patients. Results are summarized in Table 3. To the best of our knowledge, this is a comprehensive review as of November 2020. Across studies, two found a decrease GABA levels (2, 59), and three showed no significant change (54, 57, 58). However, as described in the descriptions of the individual studies and in the table, there were methodological differences in the MRS methods and treatment differences among the study populations that likely impacted the measured levels.

A decrease in GABA inhibitory action has been linked to the pathogenesis of ALS (70). GABA concentration in the extracellular space has been shown to effect cortical excitability (71). Due to GABA transporters there is a direct relationship between cellular and extracellular GABA concentrations (25, 26, 71). MRS studies in human cerebral cortex have found that the MRS GABA measurement correlates with greater cortical excitability as assessed using transcranial magnetic stimulation, frequency of seizure, and functional magnetic resonance imaging evaluation of brain activity (72).

Based on our model we anticipate that GABA and especially the GABA/glutamate ratio would be decreased in ALS patients, contributing to excessive glutamatergic activity and excitotoxicity. As such, GABA and GABA/glutamate levels in ALS patients have a potential to be a biomarker for ALS if the signal can be accurately assessed. Analyzing the ^1^H MRS spectra of GABA is more challenging due to signal overlap with other molecules, including creatine, Glx, and NAA, as well as the lower spatial resolution of the GABA measurement due to its low concentration. Therefore, in order to accurately detect the signal from GABA, spectral editing is required, as shown in detail by Shen et al. (60), although not all studies have used editing which may explain the variation in results.

To reduce the uncertainty due to methodological differences in the GABA measurement, as well as to correct for changes in neuronal volume, we also calculated the GABA/Glx ratio. Of the four studies that assessed changes in Glx, three saw a decrease in the GABA/Glx ratio, a finding in line with our hypotheses described in section A Model for Changes in MRS Measured Glutamate, GABA, and Glutamine Levels During the Progression of ALS and above. One study (59) did not measure glutamate but found reduced GABA in ALS patients relative to HCs. Thus, based on the overall findings of studies measuring glutamate, a decreased GABA/Glx ratio likely would have been observed if Glx were measured. However, as described below, limitations in the number and design of studies to date prevent a definitive conclusion from being made about the use of GABA or GABA/Glx as a biomarker for ALS.

Despite the limited studies of GABA in ALS using ^1^H MRS, there have been many published studies using this technique in other diseases (31, 73, 74). For example, a 2018 study by O'Gorman Tuura et al., 3T ^1^H MRS using MEGA-PRESS was used to evaluate GABA levels in Parkinson's patients (75). Unlike ALS, Parkinson's was predicted to be associated with an increase in GABA levels. The results of this study confirmed this hypothesis and showed a significantly higher GABA levels compared to HCs (*p* = 0.005). In addition, GABA levels correlated positively with clinical measures of disease progression (*p* = 0.038, *r* = 0.491). These results point to the potential of ^1^H MRS to detect GABA levels and act as a predictor of disease progression in a multitude of neurodegenerative diseases, including ALS, but additional studies are needed.

## Discussion

In our meta-analysis of ^1^H MRS studies to measured Glx in ALS patients, we found that the ratio of Glx/NAA was consistently greater in ALS patients relative to healthy controls, an observation consistent with the predictions of the model of increased activity of glutamatergic neurons leading to excitotoxicity and neuronal/synaptic density loss as shown by an absolute reduction in NAA levels. Although the number of studies of GABA are more limited, the ratio of GABA/Glx also showed a decrease with ALS. Both of these findings are consistent with the predictions of the model presented in section A Model for Changes in MRS Measured Glutamate, GABA, and Glutamine Levels During the Progression of ALS.

However, as discussed in section Review of ^1^H MRS Studies Evaluating Glutamate or Glx, GABA, and Glutathione in ALS, there are significant limitations in methodology and experimental design, the number of studies to date, and the Glx/NAA and GABA/Glx ratios not being directly reported in studies. In this section we focus on future directions for the application of MRS measurements of glutamate and GABA in ALS. We recommend the measurement of the Glx/NAA and GABA/Glx ratios, or other ways to normalize for neuronal loss, to both provide neuron specific measurements independent of volume changes as well as reduce methodological uncertainties. We also discuss novel developments in MRS that may allow rates of mitochondrial oxidation and neurotransmission to be measured, and their potential for more specific measurements of the pathological process than provided by metabolite concentrations.

### MRS Measurements of the Glx/NAA Ratio

In future studies, calculations of the Glx/NAA ratio ratio in ALS patients vs. healthy controls could provide insight into changes in glutamate levels, and potentially degree of hyperexcitability and associated elevated extracellular glutamate, on a per-neuron basis. As described above, the ratio with NAA has the advantage of potentially partially normalizing for partial neuronal volume loss. Further precision could be obtained by using internal referencing to unaffected regions of the brain, which will take out uncertainty due to the normal variation between healthy subjects. In addition, it takes out the difficulty in comparing studies using creatine vs. different forms of water normalization. These methodological changes would be critical to testing our hypothesis given the coefficient of variation for Glx and NAA across ALS and control groups was as high as 34% in some studies, as shown in Table 5, although the average coefficient of variation was on the order of 10–15% which is consistent with current state of the art methodology at 3 T (64).

With improved measurement precision the possibility arises that MRS of glutamate can be used to assist in the diagnosis and following of treatment in individual patients. An increase in intracellular glutamate associated with a decrease in NAA may be specific to ALS, as it has not been consistently reported in other neurodegenerative diseases that are accompanied by a decrease in NAA levels. Instead a parallel decrease is usually reported (23, 69).

An alternate explanation for elevations in the Glx/NAA ratio is that the glial glutamine level would rise due to the gliosis and other glial changes associated with inflammation, as reported by Atassi et al. (54). This would then lead to an elevated Glx at 3.0 and 1.5 T. However, based on reviews of MRS and MRSI studies of inflammatory disorders, the level of Glx is usually reduced or the same (76). This result may be explained both due to a greater drop in glutamate than an increase in glutamine, and the Glx resonance itself being weighted toward glutamate. In addition, the possibility of NAA reduction as a result of age related degeneration is acknowledged in both ALS and control groups, but unlikely given the average age across both group ranges from 42 to 65, as shown in Table 4. Within studies, the difference between the average age of ALS and control groups never exceeded 8 years.

To continue to test the hypothesis that an increase in Glx/NAA may be specific to ALS, further studies distinguishing between early vs. late stage patient populations are needed to understand how this ratio changes throughout the course of disease progression. This information would be particularly important for treatment studies to accurately assess how an intervention impacts the progression of the disease. As described in section A Model for Changes in MRS Measured Glutamate, GABA, and Glutamine Levels During the Progression of ALS and Figures 1, 2, the measured level of glutamate may reflect a competition between increases in glutamate levels in hyperexcitable glutamatergic neurons vs. a loss in their cellular density. Due to variabilities in the rate of these and other processes between individuals at baseline, the best study designs would be longitudinal studies to follow patients with early-stage ALS (e.g., defined by possible ALS) through later stages (e.g., defined by probable or definite ALS).

There have only been two published longitudinal studies using ^1^H MRS at 7.0 T to study changes in glutamate (separately from glutamine) in patients with ALS, and the findings were not consistent. Unrath et al. reported a non-significant change in glutamate levels over a 6 month period. Furthermore, NAA levels were not reported, so the Glx/NAA ratio could not be calculated. Non-significant findings may be explained by the lower 1.5 T magnitude, the small sample size, and the relatively short follow-up period. On the other hand, Cheong et al. in 2019, using a 7 T MRI system, reported a significant increase in Glx levels over a 12 month period and a greater Glx/NAA ratio in ALS patients compared to that of healthy controls, a finding that is in line with our hypothesis in section A Model for Changes in MRS Measured Glutamate, GABA, and Glutamine Levels During the Progression of ALS. Future longitudinal studies with optimum measurement of glutamate and NAA, preferably at higher magnetic fields, would help establish the specificity and sensitivity of the Glx/NAA ratio for distinguishing ALS from other neurodegenerative syndromes.

In future studies, it will also be important to maintain consistent MRS methodologies to test this hypothesis, including field strength, echo time, repetition time, voxel size, and brain region. The most frequent field strength used was 3.0 T in which 5 studies were performed. Four of these 3.0 T studies found a Glx/NAA ratio that was greater in ALS patients relative to healthy controls. In order to obtain the larger number of subjects needed to assess the value of the Glx/NAA and GABA/Glu biomarkers conclusively we suggest that future studies will most likely be conducted at 3.0 T because it is the most common field used for neuroimaging, although there are increasing number of 7.0 T systems available every year.

Across studies, other variable methodologies could explain the reported differences. For example, TE (time of echo) ranged from 5 to 68 ms, which was necessary given the range of sequencing techniques used (e.g., PRESS, STEAM, semi-LASER). It is noted that in the longer TE papers, the TE chosen allowed for partial refocusing of glutamate J modulation, giving sufficient resonance intensity.

### MRS Measurements of the GABA/Glx Ratio

The imbalance between excitatory neurotransmitters (Glx) and inhibitory neurotransmitters (GABA) is important in the pathogenesis of ALS and a range of other psychiatric and neurological disorders (2, 77). As described in section A Model for Changes in MRS Measured Glutamate, GABA, and Glutamine Levels During the Progression of ALS, GABA release from interneurons, both *via* neurotransmission and reverse transport, has a strong influence on the excitability of glutamatergic neurons. Therefore, in principle the GABA/Glx measurement in combination with the Glx/NAA ratio may provide both diagnostic insight into distinguishing early ALS from other disorders as well as monitoring of GABAergic targeted treatments. The GABA/Glx ratio also normalizes for NAA concentration, as it is equivalent to GABA/NAA divided by Glu/NAA, which helps address the uncertainty in the absolute concentration of metabolites as shown in Table 4.

The combination of a decrease in the GABA/Glx ratio with an elevated Glx/NAA ratio may enhance the specificity for detection of ALS. Although changes in the GABA/Glx ratio have been reported in other diseases (73, 78–80), association with decreased NAA and increased Glx/NAA may be specific to ALS. In order to test this hypothesis longitudinal studies will be needed as the ratios may be dependent on disease progression. As similarly stated about Glx/NAA, we suggest that future studies are conducted at 3.0 T given the greater availability of the technology in a clinical setting in order to test this hypothesis in a large patient population. In our sub-analysis, three out of the four studies at 3.0 T showed an increase in the GABA/Glx ratio.

### MRS Measurements of Glutathione to Assess Enhanced ROS Production

Glutathione is a metabolite that is synthesized from glutamate and can be detected using ^1^H-MRS. As described in section A Model for Changes in MRS Measured Glutamate, GABA, and Glutamine Levels During the Progression of ALS the most common idiopathic forms of ALS are associated with overproduction of ROS due to mutations in superoxide dismutase (SOD) which result in overproduction of the ROS hydrogen peroxide (H_2_O_2_). Glutathione is an important compound in the chemical reduction and inactivation of ROS. A decline in the rate of ROS reduction, reflected by a decrease in glutathione levels, has been proposed to be an important mechanism in damage and neurodegeneration. Although this review primarily focuses on Glx and GABA, recent improvements in MRS spectral editing methodology has led to measurements of brain glutathione levels in a number of neurological disorders such as Parkinson's disease (81) and Alzheimer's disease (82).

There have been two published studies on changes in glutathione levels in ALS patients relative to healthy controls, but results were inconsistent (55, 83). In addition, it is not known whether glutathione is decreased early in the progression of ALS, and therefore may play a role in accelerating the disease process, or alternatively decreases due to cell damage as the disease progresses. Further studies, particularly longitudinal studies, are needed to better understand the relationship between glutathione and disease progression in ALS. Reliable studies will need to use spectral editing to determine glutathione levels due to the overlap of the spectrum with other metabolites.

### Predictions About MRS Measurements of Glx/NAA and GABA/Glx in the Presence of Glutamatergic System Targeted Drug Treatments

There are currently multiple pre-clinical research studies and clinical trials evaluating the treatment of ALS with of drugs targeting the glutamatergic (84) and GABAergic systems (85). At the time of this publication two drugs targeted at the glutamatergic system, Riluzole and Edaravone, are the only FDA approved drugs to slow the progression of ALS. Riluzole acts to reduce the release of glutamate from glutamatergic neurons (*via* inhibition of Ca^2+^ channels) and increasing re-update of glutamate by astrocytes (86, 87). Edaravone prevents reactive oxygen species from being formed and/or removes them before they can damage neurons, in effect limiting apopotosis of post synaptic terminals as well as the inactivation of EAAT2 receptors in astrocytes and increasing the rate of glutamate re-uptake (Figure 1).

Based on these pharmacological effects, we have summarized in Table 6 our predictions on how MRS detected changes in ALS patients taking Riluzole and Edaravone may compare to ALS patients not taking these drugs. In both cases we assumed that the primary impact of the medication will be too reduce synaptic and extracellular glutamate levels is by enhancing glial glutamate uptake. As shown in Figure 1, enhancing glial uptake will prevent the rise in synaptic and extracellular glutamate associated with hyperexcitability. Therefore, the rise in extracellular glutamate concentration associated with higher signaling activity will be blunted. Furthermore, the reduced extracellular glutamate will slow down the excitotoxicity process and resultant neuronal damage and loss. We thus predict that for both drugs, and for drugs targeted at reducing extracellular glutamate levels in general, there will be lower increases in the Glx/NAA ratio as well as reduced reductions in NAA levels associated with neuronal volume loss (Table 6). If given sufficiently early these drugs may lengthen the time before these MRS changes develop, and the underlying pathology that gives rise to them.

**Table 6:** Predictions on MRS detected changes over time in ALS patients taking Riluzole and Edaravone, both of which target enhancing astrocytic glutamate uptake and therefore lowering synaptic and extracellular glutamate levels, relative to ALS patients not taking these drugs.

**Example**	**Class of drugs**	**Impact to synaptic glutamate**	**Impact to Glx/NAA**	**Impact to GABA/Glx**	**Impact to NAA**
Riluzole	Benzothiazoles	Decrease	Reduced rate of increase	Reduced rate of decrease	Reduced rate of decrease
Edaravone	Antioxidants	Decrease	reduced rate of increase	Reduced rate of decrease	Reduced rate of decrease

As described in section Review of ^1^H MRS Studies Evaluating Glutamate or Glx, GABA, and Glutathione in ALS, several MRS studies have included ALS patients being treated with Riluzole (2, 59) and did not report conclusive differences. However, given the variation between patients in stage of disease, as well as the variation among healthy subjects in glutamate and GABA levels (absolute and relative to NAA), it is likely that longitudinal studies will be needed to detect changes.

In clinical trials of candidates for ALS therapeutics, MRS measurements of Glx/NAA and GABA/Glx could act as novel outcome measures for comparison of efficacy in placebo groups vs. treatment groups. Typical outcome measures in ALS clinical trials currently focus on survival and on clinician-assessed measurements of function, namely ALSFRS-R scores. However, these outcome measures have limitations: survival outcomes do not provide real-time results on the efficacy of drugs and may be influenced by other co-morbid factors; clinician-assessed measurements of functions are subjective and may not be reliable indicators in trials of early-stage ALS patients. MRS measured ratios of Glx/NAA and GABA/Glx, on the other hand, are unbiased, are more likely to detect changes in early-stage ALS patients, and can be obtained frequently to understand drug efficacy faster. These ratios therefore may provide more insight into drug efficacy during Phase I and II clinical trials, in effect reducing the risk of failed candidates in Phase III trials (88).

### MRS Measurements of Mitochondrial Energy Production and Glutamate/GABA/Glutamine Cycling

As described in section A Model for Changes in MRS Measured Glutamate, GABA, and Glutamine Levels During the Progression of ALS, in ^1^H-MRS the level of glutamate is used as a biomarker for the flux through the glutamate/GABA/glutamine cycle. Although studies support that there is a positive relationship between concentration and flux, there are conditions in which this relationship may be altered. In parallel with the development of ^1^H-MRS methods, other MRS methods measuring active stable (non-radioactive) isotopes, including ^13^C and ^2^H, have been developed that specifically measure flux and mitochondrial energy production. More specifically, these methods can be used to measure V_TCA_, the rate of the TCA cycle in neurons and astrocytes, as well as V_cycle−glu_, the rate of conversion of glutamate to glutamine in astrocytes. For detailed descriptions see the following reviews and references therein (15, 89–92).

Based on studies in which both ^13^C MRS measurements of fluxes and ^1^H-MRS measurements of glutamate concentration have been performed, it is likely that the ^13^C (and ^2^H) MRS methods will also be more sensitive for picking up changes in V_TCA_ and V_cycle−Glu._ Evidence for the higher sensitivity is from clinical studies of riluzole in rats undergoing chronic unpredictable stress, which leads to hippocampal chronic excitability and loss of synaptic density which also occurs in ALS. In these studies, application of riluzole during the stress period blocked large reductions in rates of the TCA cycle and glutamate/GABA/glutamine cycling which were detected by ^13^C MRS (86), in contrast to smaller changes in glutamate and GABA concentrations. Similarly recent studies in human frontal lobe of the glutamatergic drug ketamine have shown a larger change in V_cycle−glu_ than changes in glutamate and glutamine concentration (93, 94). However, these advantages come at the cost of considerably more demanding technological and cost requirements (15), as well as the further disadvantage that the ability to perform these measurements is only available at specialized MRI centers. However, the recent development of DMI (deuterium metabolic imaging) promises to make these measurements more widely available as it can easily be performed on modified clinical MRI systems (89).

### Conclusions and Recommendations

^1^H-MRS may provide the unique ability to reveal biomarkers that are specific to ALS and sensitive enough to diagnose patients at early stages in disease progression. Measuring absolute concentrations of glutamate and GABA, metabolites associated with the pathogenesis of ALS, may yield variable results. However, measuring these metabolites as normalized ratios, Glx/NAA and GABA/Glx, reduces methodological uncertainties and provides measurements on a per-neuron basis that are independent of neuronal volume changes. Future studies, however, are needed to confirm these hypotheses. To conclude this review, we have summarized our overall recommendations as follows.

#### Suggested Methodology for ^1^H-MRS Measurements of Glutamate and GABA

- Calculations should be normalized to account for uncertainties in absolute concentration measurements, and for changes in neuronal volume. We suggest presenting these ratios as: Glx/NAA and GABA/Glx, or glutamate/NAA and GABA/glutamate
- Greater precision may be obtained using internal referencing to unaffected regions of the brain, as well as high field strength (e.g., 7.0 T). These methods will allow for greater resolution of glutamate and glutamine, allowing ratios to presented in the form of glutamate rather than Glx.
- In general, we recommend following the recommendations in the ^1^H MRS methodology consensus statements cited in section A Model for Changes in MRS Measured Glutamate, GABA, and Glutamine Levels During the Progression of ALS.

#### Possible Clinical Applications for ALS Patients

- Greater certainty in diagnosis of ALS, in combination with other clinical measures (e.g., ALSFRS-R score)
- Faster diagnosis of early-stage ALS, allowing for a decreased time period between symptom onset and initiation of treatment
- Monitoring of disease progression over time
- Un-biased efficacy assessment of approved therapeutics (Edaravone and Riluzole) and pre-clinical/clinical therapeutics that work to reduce extracellular glutamate
- Real-time results on the efficacy of pre-clinical and clinical therapeutics, leading to faster screenings of early stage candidates and reductions in failed late-stage candidates.

#### Future Directions

- Additional ^1^H-MRS studies with similar acquisition strategies, patient and control populations, and sequenced brain regions are needed to confirm stated hypotheses about Glx/NAA and GABA/Glx ratios. Full datasets for each study are also needed to understand the variance in the Glx/NAA and GABA/Glx ratios.
- Additional ^1^H-MRS studies using higher magnetic field strengths and optimal measurements of metabolite concentrations and ratios are needed to establish specificity and sensitivity of the Glx/NAA and GABA/Glx ratios for ALS
- Longitudinal studies are needed to understand how these ratios change throughout ALS disease progression and compare to patients with other neurological syndromes
- Greater volumes of studies measuring glutathione are needed to better understand the relation to disease progression in ALS
- ^13^C MRS and DMI studies in ALS patients could provide greater sensitivity for changes in mitochondrial energetics through measurement of metabolite flux (V_TCA_ and V_cycle−Glu_).

## Author Contributions

SC wrote the first draft of the manuscript with input from DR. DR and SC developed the model and its predictions regarding changes in metabolite levels in ALS. SC did the primary literature review with assistance from DR. All authors contributed to the writing of the manuscript drafts and approved the submitted version.

## Conflict of Interest

The authors declare that the research was conducted in the absence of any commercial or financial relationships that could be construed as a potential conflict of interest.

## Publisher's Note

All claims expressed in this article are solely those of the authors and do not necessarily represent those of their affiliated organizations, or those of the publisher, the editors and the reviewers. Any product that may be evaluated in this article, or claim that may be made by its manufacturer, is not guaranteed or endorsed by the publisher.
